# A Novel HA/β-TCP-Collagen Composite Enhanced New Bone Formation for Dental Extraction Socket Preservation in Beagle Dogs

**DOI:** 10.3390/ma9030191

**Published:** 2016-03-11

**Authors:** Ko-Ning Ho, Eisner Salamanca, Kuo-Chi Chang, Tsai-Chin Shih, Yu-Chi Chang, Haw-Ming Huang, Nai-Chia Teng, Che-Tong Lin, Sheng-Wei Feng, Wei-Jen Chang

**Affiliations:** 1 School of Dentistry, College of Oral Medicine, Taipei Medical University, Taipei 110, Taiwan; d204097003@tmu.edu.tw (K.-N.H.); eisnergab@hotmail.com (E.S.); newdent.clinic@msa.hinet.net (T.-C.S.); ivytainan@hotmail.com (Y.-C.C.); dianaten@tmu.edu.tw (N.-C.T.); chetong@tmu.edu.tw (C.-T.L.); b8702070@tmu.edu.tw (S.-W.F.); 2 Department of Chemical Engineering and Biotechnology, National Taipei University of Technology, Taipei 106, Taiwan; hu2637@yahoo.com.tw; 3 Graduate Institute of Biomedical Materials & Tissue Engineering, College of Oral Medicine, Taipei Medical University, Taipei 110, Taiwan; hhm@tmu.edu.tw; 4 Dental Department, Taipei Medical University Hospital, Taipei 110, Taiwan; 5 Dental Department, Taipei Medical University, Shuang-Ho Hospital, New Taipei 235, Taiwan

**Keywords:** hydroxyapatite, β-tricalcium phosphate, composite, collagen, allograft, socket preservation, animal study

## Abstract

Past studies in humans have demonstrated horizontal and vertical bone loss after six months following tooth extraction. Many biomaterials have been developed to preserve bone volume after tooth extraction. Type I collagen serves as an excellent delivery system for growth factors and promotes angiogenesis. Calcium phosphate ceramics have also been investigated because their mineral chemistry resembles human bone. The aim of this study was to compare the performance of a novel bioresorbable purified fibrillar collagen and hydroxyapatite/β-tricalcium phosphate (HA/β-TCP) ceramic composite *versus* collagen alone and a bovine xenograft-collagen composite in beagles. Collagen plugs, bovine graft-collagen composite and HA/β-TCP-collagen composite were implanted into the left and right first, second and third mandibular premolars, and the fourth molar was left empty for natural healing. In total, 20 male beagle dogs were used, and quantitative and histological analyses of the extraction ridge was done. The smallest width reduction was 19.09% ± 8.81% with the HA/β-TCP-collagen composite at Week 8, accompanied by new bone formation at Weeks 4 and 8. The HA/β-TCP-collagen composite performed well, as a new osteoconductive and biomimetic composite biomaterial, for socket bone preservation after tooth extraction.

## 1. Introduction

The alveolar process forms during tooth eruption and undergoes atrophy after the loss of single or multiple teeth [1,2]. Generally, after tooth removal, resorption of the buccal compartment of the ridge is more pronounced than that of the lingual/palatal portion [3]. In the post-extraction healing phase, the alveolar bone undergoes additional atrophy as a result of natural remodeling [4,5]. After tooth extraction, resorption of up to 50% of the alveolar ridge (AR) width can occur in 12 months. Resorption at extraction sockets often creates a residual knife edge [6]. The estimated structural loss is as much as 40% and 60% of the pre-extraction AR height and width, respectively [7]. This loss has a detrimental effect on potential treatment with dental implants or conventional prostheses. Ideally, sufficient vertical and horizontal alveolar bone volume must be preserved at the site of implant insertion or prosthesis loading, AR preservation becoming important for successful restorative treatments [8]. Therefore, limiting consequent ridge resorption is important after tooth extraction.

There are many techniques available to improve alveolar ridge collapse after tooth extraction, including connective tissue grafts, acellular dermal matrix grafts and guided bone regeneration with or without the association of different bone grafts [9]. This procedure is called socket augmentation or socket preservation, where also the use of bone biomaterials has been advocated to augment this extraction sites in conjunction with implant placement to support bone-implant contact during integration [10]. All grafts aim to enhance wound healing and to preserve as much bone as possible. One key factor is to understand the natural process of healing in sockets [11]. Another key factor is angiogenesis, the process by which new capillaries grow from pre-existing mature capillaries (*i.e.*, from resident endothelial cells) by sprouting, intussusception or elongation [12]. Angiogenesis is a complex, multistep process, comprising extracellular matrix (ECM) degradation, cell proliferation, and migration. Therefore, for bone tissue engineering, the scaffold design must mimic the structure and properties of bone ECMs, incorporating elements, such as harvested cells, recombinant signaling molecules and three-dimensional (3D) matrices, simultaneously preserving the clot and promoting angiogenesis, to generate bone tissue with good functional and mechanical qualities [13,14,15].

The most widely-used xenogeneic bone substitutes are sintered bovine bone with a 3D matrix and osteoconductive bone substitute materials. Bone substitute materials exhibit excellent hard tissue biocompatibility despite their low degradation rate [16] and have a crystalline and morphologic structure similar to human cancellous bone. The porous nature of the bioactive bone substitute Bio-Oss collagen, which comprises 75% of the overall volume of the material, greatly increases its surface area [17]. This increased surface area enhances angiogenesis and serves as a scaffold for new bone formation.

Type I collagen is an important 3D structural component of tissues and the main constituent of the ECM of bone tissue. It represents an excellent delivery system for growth factors and facilitates the migration and penetration of osteoblasts and vessels, thus promoting angiogenesis and new bone formation [16]. Therefore, the addition of type I collagen to bone substitutes may preserve the clot and support angiogenesis. A mixture of type I collagen and sintered bovine bone formed a 3D structure with better clot preservation and angiogenesis, showing alveolar bone preservation and enhanced bone tissue engineering [16].

The most common synthetic alloplastic biomaterials currently in use are calcium phosphate-based (Ca-P) bioceramics. Ca-P ceramics have been extensively investigated because their mineral chemistry resembles that of human bone [18]. Ca-P ceramics have a composition and structure similar to the mineral phase of bones and are osteoconductive [19]. Ca-P ceramics are also commonly used in medical applications because of their biocompatibility, safety, unlimited availability and cost effectiveness [20].

In the previous study, Ca-P ceramics also performed better on new bone formation in the rabbit calvaria [19]. However, Ca-P bioceramics also have some disadvantages: poor mechanical properties, time-consuming fabrication, low product yield, lack of an organic phase, impurities, nonhomogeneous particle size and shape, large grain size and difficult porosity control [21]. These limitations have triggered further investigations and development of other synthetic materials, such as polymeric cyanoacrylate composite [21]. Furthermore, some researchers in the past few years have focused on the design and development of 3D-printed advanced scaffolds, which offer significant advantages [22,23,24]. A combination of Ca-P bioceramics can support the bone plate around the tooth extraction socket, and collagen can be a key factor for angiogenesis. In combination, both materials were considered optimal for socket preservation; therefore, in the present study, a novel composite for extraction socket preservation, freeze-dried hydroxyapatite/β-tricalcium phosphate (HA/β-TCP) ceramic mixed with homogenous collagen was developed.

The aim of this study was to compare the performance of a novel bioresorbable purified fibrillar collagen and HA/β-TCP ceramic composite *versus* collagen alone and a bovine xenograft-collagen composite at the time of enhancing dental extraction socket preservation in beagle dogs.

## 2. Results

### 2.1. Scanning Electron Microscope Examination

**Figure 1:**
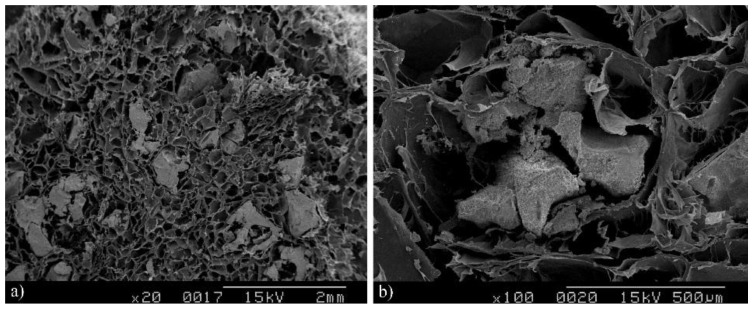
SEM structure of hydroxyapatite/β-tricalcium (HA/β-TCP) phosphate-collagen composite. The scanning electron microscope photograph showed the HA/β-TCP granules to be homogenous (**a**) (magnification 20×) and integrated in the collagen matrix (**b**) (magnification 100×).

The scanning electron microscope examination showed the HA/β-TCP granules homogenously distributed with the collagen matrix (Figure 1a). The collagen matrix also demonstrated integrity with the surrounding HA/β-TCP granules (Figure 1b).

### 2.2. Energy Dispersive Spectrometry

**Figure 2:**
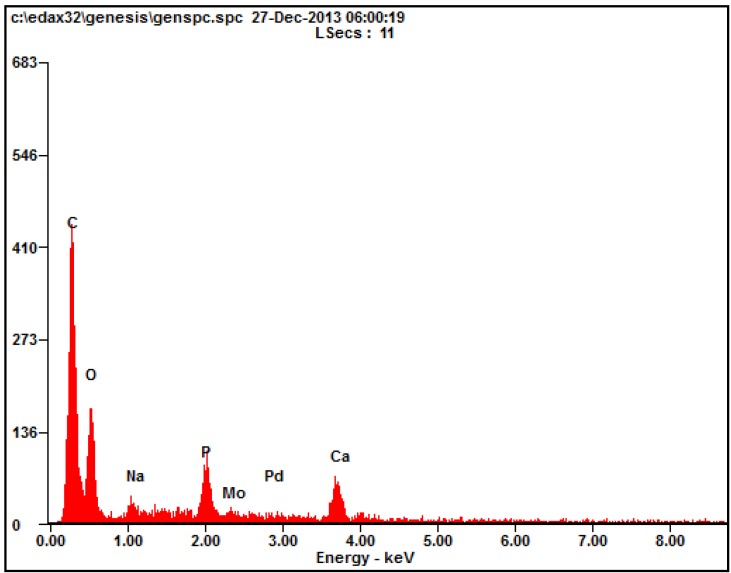
Energy dispersive spectrometry (EDS) of the hydroxyapatite/β-tricalcium phosphate-collagen composite.

The energy dispersive spectrometry (EDS) analyses of the HA/β-TCP collagen composite showed a chemical analysis composition with a calcium (Ca)/phosphate (P) molar ratio of 1.83 (Figure 2).

### 2.3. Energy Dispersive Spectrometry with Element’s Weight and Atomic Percentages: High Carbon Levels belong to the Collagen, while the Ca/P Molar Ratio Was 1.83: Compression Strength

The compression strength of (HA/β-TCP)-collagen composites was examined. The results showed maximum displacement of 10.226 ± 0.021 mm. The compression strength was 0.545 ± 0.019 MPa.

### 2.4. Cell Vitality and Biocompatibility

**Figure 3:**
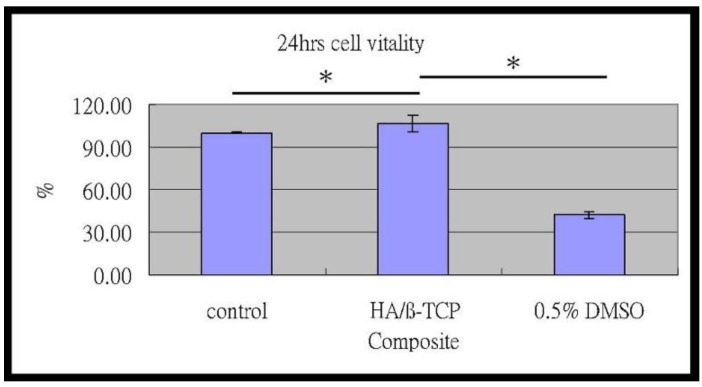
Cell vitality.

The MTT assay showed that, after 24 h of culture, the numbers of MG-63 cells with the composite media were greater than the control group. Besides, the cells cultured with composite extraction demonstrated a significantly higher cell vitality rate in comparison to the positive and negative control group (p < 0.01) (Figure 3).

The MTT assay of MG-63 cells: compared to the control group, the HA/β-TCP-collagen composite was significantly greater after 24 hours of culture (** *p* < 0.01).

### 2.5. Dimensional Changes of the Alveolar Bone

**Figure 4:**
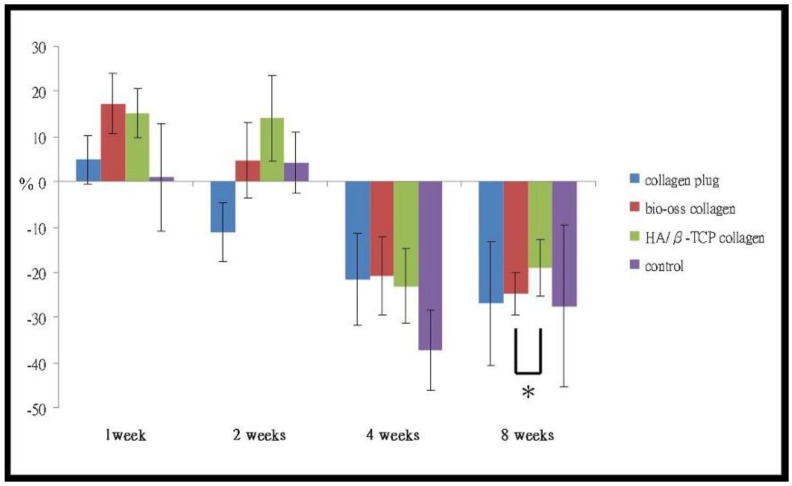
Horizontal dimensional change.

The horizontal dimension increased in Week 1 in all groups, the control group having a slight increase of 1.03% ± 13.73% and bovine graft-collagen showing a major increase in the alveolar ridge with 17.36% ± 6.54%. The HA/β-TCP-collagen composite was close to the bovine graft-collagen group with 15.10% ± 10.21%. The collagen plug group showed a 4.89% ± 5.40% increase, behaving more like the control group. The vertical dimension decreased in all groups. The collagen plug group decreased less with −0.16 ± 0.15 mm and the HA/β-TCP-collagen composite group the most with −0.33 ± 0.13 mm. The control group with −0.31 ± 0.12 mm decreased in a similar way to the HA/β-TCP-collagen composite, and the bovine graft-collagen group decreased only −0.27 ± 0.07 mm. The detailed horizontal dimensional changes of the alveolar bone that occurred at the sites of graft implantation appear in Figure 4 and Table 1. Table 2 demonstrates the vertical dimensional changes of the alveolar bone that occurred at the sites of graft implantation.

Horizontal dimensional changes occurred in the percentage of the alveolar bone implanted with collagen, bovine xenograft-collagen composite or hydroxyapatite/β-tricalcium phosphate (HA/β-TCP)-collagen composite in Weeks 1, 2, 4 and 8. Significant differences were evident between groups implanted with the bovine xenograft-collagen composite and HA/β-TCP-collagen composite at Week 8 (* *p* < 0.01).

At Week 2, a horizontal decrease of −11.14% ± 6.72% was noticed in the collagen plug group. The rest of the groups showed a horizontal increase of the AR, the HA/β-TCP-collagen composite group with 13.99% ± 8.52% being the highest increase, followed by the bovine graft-collagen and control groups with 4.71% ± 8.33% and 4.20% ± 4.70%, respectively. There was a vertical dimension decrease in all of the groups. The collagen plug group decreased less, with −0.24 ± 0.17 mm, followed by −0.29 ± 0.12 mm for the control group. The HA/β-TCP-collagen composite group decreased the most with −0.38 ± 0.08 mm. The bovine graft-collagen group had the second highest vertical reduction (−0.37 ± 0.07 mm).

The control group suffered the highest with a −37.29% ± 6.26% horizontal decrease during Week 4. At this time, the collagen plug, bovine graft-collagen and HA/β-TCP-collagen composite groups decreased their horizontal dimension in a similar way with −21.63% ± 5.50%, −20.87% ± 9.60% and −23.10% ± 8.20%, respectively. The vertical dimension of all of the groups decreased at Week 4. The control group had the highest decrease with −0.34 ± 0.12 mm, followed by the HA/β-TCP-collagen composite and bovine graft-collagen groups with −0.41 ± 0.24 mm and −0.37 ± 0.16 mm, respectively. The collagen plug group with −0.29 ± 0.21 mm was the lowest vertical reapportion.

A greatest loss in the horizontal dimension occurred in Week 8. The smallest horizontal dimensional reduction of the alveolar ridge in Week 8 was −19.09% ± 8.81% in the HA/β-TCP-collagen composite group, followed by −24.82% ± 6.90% in the bovine graft-collagen composite group. A −26.93% ± 11.89% horizontal reduction of the alveolar ridge with the collagen plug group was reached and close to a −27.47% ± 17.94% reduction reached for the control group. The highest vertical dimensional reduction of the alveolar bone in Week 8 was −0.41 ± 0.24 mm for the HA/β-TCP-collagen composite group followed by −0.37 ± 0.16 mm for the bovine graft–collagen group. The control group with a −0.34 ± 0.12 mm behaved in a similar way to the collagen group; this last group suffered the smallest vertical dimensional change of the alveolar bone at Week 8 (−0.29 ± 0.21 mm).

### 2.6. Histological and Histomorphometry Analysis

**Figure 5:**
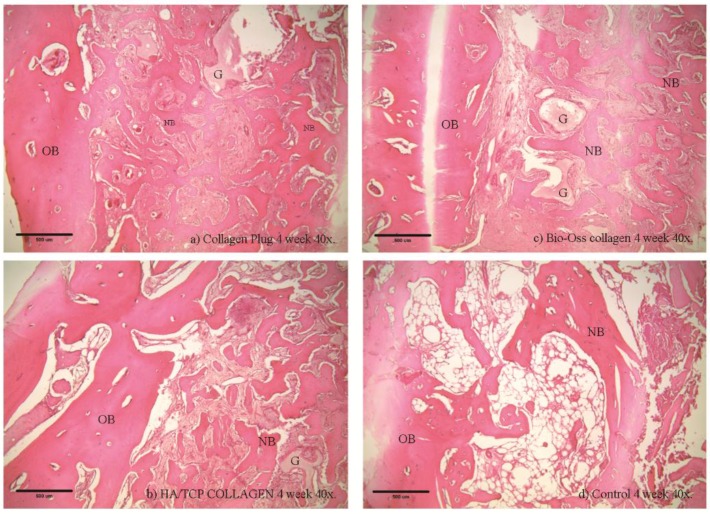
Histological examination at the fourth week using 40× magnification Histological analysis of the collagen (**a**), bovine xenograft-collagen composite (**b**), hydroxyapatite/β-tricalcium phosphate (HA/β-TCP)-collagen composite (**c**) and control groups (**d**). Hematoxylin and eosin staining; original magnification: 40×. Abbreviations: NB, newly-formed bone; OB, old bone; G, granule residual.

**Figure 6:**
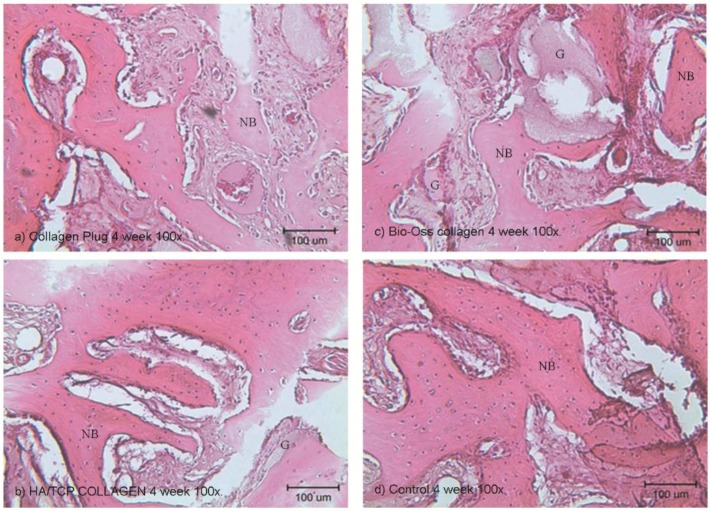
Histological examination at the fourth week using 100× magnification. Histological analysis of the collagen (**a**), bovine xenograft-collagen composite (**b**), hydroxyapatite/β-tricalcium phosphate (HA/β-TCP)-collagen composite (**c**) and control groups (**d**). Hematoxylin and eosin staining; original magnification: 100×. Abbreviations: NB, newly-formed bone; G, granule residual.

**Figure 7:**
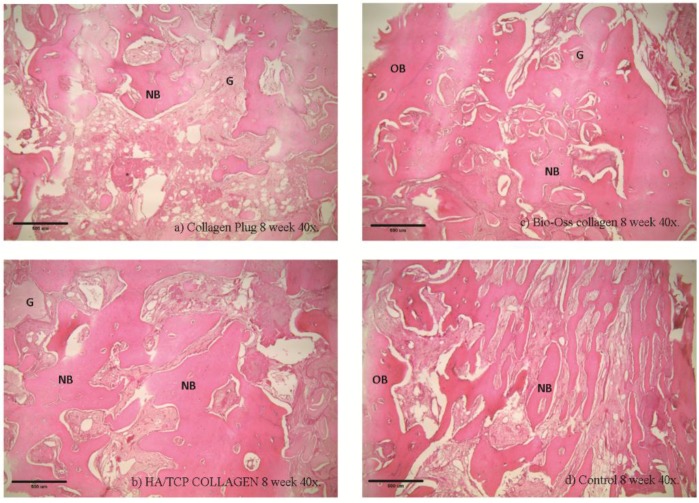
Histological examination at the eight week using 40× magnification. Histological analysis of the collagen (**a**), bovine xenograft-collagen composite (**b**), hydroxyapatite/β-tricalcium phosphate (HA/β-TCP)-collagen composite (**c**) and control groups (**d**). Hematoxylin and eosin staining; original magnification: 40×. Abbreviations: NB, newly-formed bone; OB, old bone; G, granule residual.

**Figure 8:**
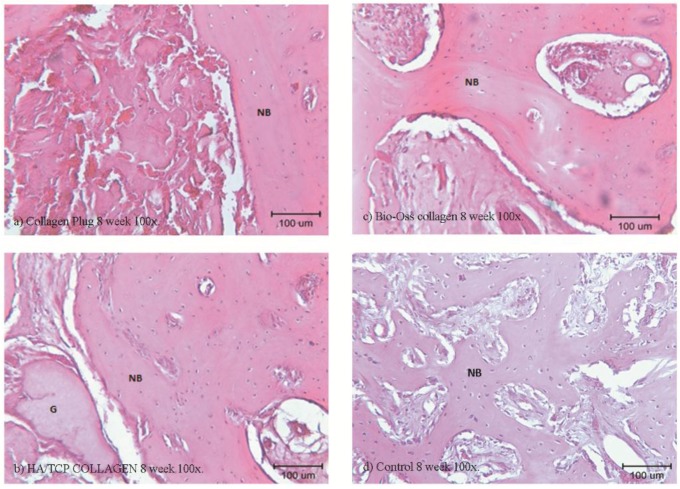
Histological examination at the eight week using 100× magnification. Histological analysis of the collagen (**a**), bovine xenograft-collagen composite (**b**), hydroxyapatite/β-tricalcium phosphate (HA/β-TCP)-collagen composite (**c**) and control groups (**d**). Hematoxylin and eosin staining; original magnification: 100×. Abbreviations: NB, newly-formed bone; G, granule residual.

Histological examinations were performed in Week 4 (Figure 5 and Figure 6) and Week 8 (Figure 7 and Figure 8). As shown in (b) and (c) of the four figures, the HA/β-TCP-collagen and bovine graft-collagen composites were visible in both Weeks 4 and 8; however, collagen plugs were slightly more degraded in the same time period. Furthermore, in the same figures can be seen numerous blood vessels colonizing the granulation tissue, and calcified osteoid tissues were evident around all of the materials at Weeks 4 and 8. New bone was deposited over the inorganic matrix provided by the implanted composite biomaterials. The composite materials were surrounded by an osteoid border, which started at the metaphyseal tissue and continued growing in close contact with the implanted materials. New bone formation was homogeneous, progressive and centripetal with all implanted materials.

Remodeling and calcification of the newly-formed bone were evident. Well-connected trabeculae surrounding the degraded materials were visible in Week 8 (Figure 7 and Figure 8). Nearly mature bone, which was less cellular, more mineralized and structurally better organized into the lamellar bone, was noted after implantation of the HA/β-TCP-collagen composite, as shown in Figure 8b.

**Figure 9:**
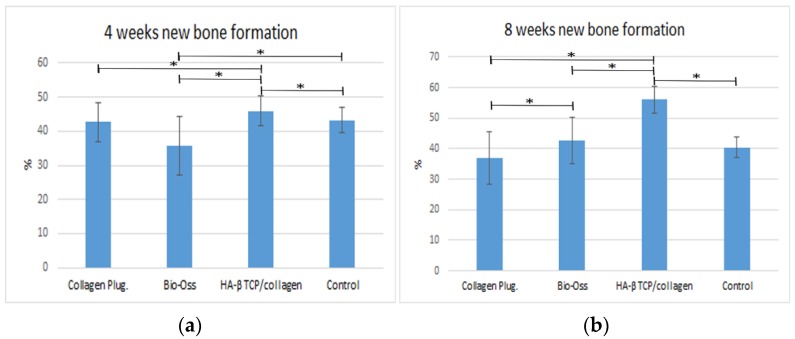
New bone formation. Average of new bone formation (bone volume/tissue volume) at four (**a**) and eight weeks (**b**). * *p* < 0.05.

Bone regeneration was observed from the walls of the sockets in all of the tested materials, serving as scaffolds for the growth of new bone. Histomorphometry analysis at four weeks showed on average 43.3% ± 3.76% (*p* < 0.05) new bone in the control group; 45.95% ± 4.41% new bone formation in the HA/β-TCP-collagen composite group was observed, with *p* < 0.05 in relation with the 35.6% ± 8.58% of new bone in the bovine graft group. The collagen plug group had 42.72% ± 5.75% new bone formation, which did not present a statistical difference within the bovine graft group and control group (Figure 9).

At eight weeks was found the highest new bone formation with 56.10% ± 4.41% in the HA/β-TCP-collagen composite group (*p* < 0.05). Lower new bone formation of 36.94 ± 8.68% was in the collagen plug group; 42.68% ± 7.46% new bone was found in the bovine graft group (*p* < 0.05) in relation to the collagen plug group. The control group presented 40.43% ± 3.26% new bone (Figure 9).

Signs of biomaterial resorption, such as phagocytic cells forming Howship’s lacunae, etching, pits and resorptive trail formation, were identified on the granules’ surface of the bovine xenograft-collagen composite and HA/β-TCP-collagen composite groups. The collagen in all of the materials served as a framework upon which the new bone was generated and later spread. From this observation, we infer that after Week 4 of the implantation in rabbits’ calvariae, all materials showed osteoconductive properties, with no statistically-significant difference between them and the control group. At Week 8, despite the presence of the material granules, resorption signs were visible.

## 3. Discussion

Preclinical and clinical studies have demonstrated that alveolar ridge volume loss post extraction is an irreversible process that involves both horizontal and vertical reduction [25]. In the search for a synthetic biomaterial able to reduce the post-extraction bone loss and at the same time replace the autograft, a range of materials has been developed over the past four decades. Various calcium/phosphate biomaterials, which resemble either the composition of bone mineral or its precursors, have been developed, such as HA-, α- and β-tricalcium phosphate, octa-calcium phosphate and di-calcium phosphate, in the form of ceramics, cements and thin coatings [24,26,27]. Many relatively insoluble Ca-P materials are osteoconductive and, in some cases, able to induce new bone formation at extra skeletal sites [26,28,29,30,31].

The first successful application of a Ca-P reagent [32] in humans was reported in 1920 [33]. The first use of TCP in surgically-created periodontal defects in animals was documented 50 years later [34]. The application of dense HA has also been reported as an immediate tooth root replacement [35]. Synthetic HA and β-TCP have been commercially available as bone graft substitute materials in the medical and dental fields for the last three decades [36,37]. Biphasic calcium phosphate (BCP) is a bioactive ceramic comprising two mixed phases: the less soluble HA and the more soluble β-TCP; its chemical properties depend on the HA/β-TCP ratio [38]. In this study, the ratio of collagen bone graft matrix to HA/β-TCP was 30:70, showing in the EDS the collagen with HA/β-TCP granules, a calcium/phosphate molar ratio of 1.83. SEM displayed a structure with pores and a rough surface characteristic of a scaffold material that enhances cell proliferation, as was possible to see with the vitality analysis, where cell vitality was improved by the presence of the material.

After tooth extraction, the vertical dimensions of AR were moderately reduced in all groups. No statistically-significant difference was evident between grafted and non-grafted sites. Therefore, neither collagen plugs, bovine graft-collagen composite nor HA/β-TCP-collagen composite preserved the vertical dimensions of ARs. The vertical reabsorption in all of the groups may be related to the material´s vertical mechanical strength and appears to be insufficient to hold the vertical dimension of ARs.

The horizontal dimensions of the extraction sites increased in Week 1 for all of the groups. There was a moderate increase of the alveolar ridge in Week 2 for the bovine graft-collagen composite and control groups. At two weeks, the HA/β-TCP-collagen composite group showed the highest increased alveolar ridge (Figure 4). The alveolar ridge horizontal increase suffered in all of the groups after extraction in Week 1, and initiation of wound healing in Week 2 can be related to the acute inflammation suffered by the dental socket post-extraction. Subsequently, in Weeks 4 and 8, the horizontal dimensions of the extraction sites were preserved when implanted with collagen plugs, bovine graft-collagen composite and HA/β-TCP-collagen composite. Therefore, collagen plugs, bovine graft-collagen composite and HA/β-TCP-collagen composite preserve the horizontal dimensions of ARs. Furthermore, preservation of the horizontal dimensions of the alveolar bone was more significant in the groups where the HA/β-TCP-collagen composite was implanted, followed by the bovine graft-collagen composite and in third place the collagen plug, the control groups having the most horizontal resorption. Not all of the horizontal measurements present statistically-significant differences, except for the HA/β-TCP-collagen composite and bovine graft-collagen groups at Week 8. The HA/β-TCP-collagen composite facilitated the best horizontal dimensional preservation at the extraction site, followed by the bovine graft-collagen.

For effective preservation of AR, the primary consideration must be angiogenesis, with bone regeneration to prevent socket wall collapse as the secondary consideration. Thus, osteoconduction by the scaffold is necessary. Collagen plugs containing type I collagen can serve as scaffolds and assist the migration and penetration of osteoblasts and vessels to promote angiogenesis [16]. On the other hand, the bovine graft-collagen and HA/β-TCP-collagen composites exhibit both osteoconductive 3D scaffolding and angiogenic abilities. They allow better AR preservation than collagen plugs, which only preserve the clot for angiogenesis.

Our histological analysis revealed remodeling and calcification of the newly-formed woven bone, and well-connected trabeculae were visible surrounding the degraded materials in Week 8. Nearly mature bone, which was less cellular, more mineralized and structurally better organized into the lamellar bone, was present after implantation of the HA/β-TCP-collagen composite, as shown in Figure 5b. Because the lamellar bone is better at load bearing and impact resistance than the woven bone [39], the HA/β-TCP-collagen composite demonstrates similar bone regeneration qualities compared to the other biomaterials and was faster than the control group in this study.

It is established that inflammation is a significant element of the host response to biomaterials and, as such, is used as a measure of biocompatibility in both acute and chronic periods. Osteoclasts and macrophages are two cell types comprising the major components of the cellular host response that cause the formation of a fibrous sheath in place of viable bone tissue [40]. Previous studies have shown that β-TCP granules induce controlled levels of membrane lysis through dissolution of their surfaces. This effect results in high phosphate and low calcium levels, increasing macrophage adhesion and sustaining viable osteoblast adhesion [41]. Previous studies have also proven that highly purified β-TCP provides early bone conduction, followed by its resorption and replacement with the newly-formed bone [42]. Accordingly, we suggest that the HA/β-TCP-collagen composite, which exhibits good 3D scaffold properties based on the limited degradation of HA and good angiogenic ability added by the collagen and resorption of β-TCP, can be a bone graft substitute for socket preservation following tooth extraction.

## 4. Materials and Methods

**Figure 10:**
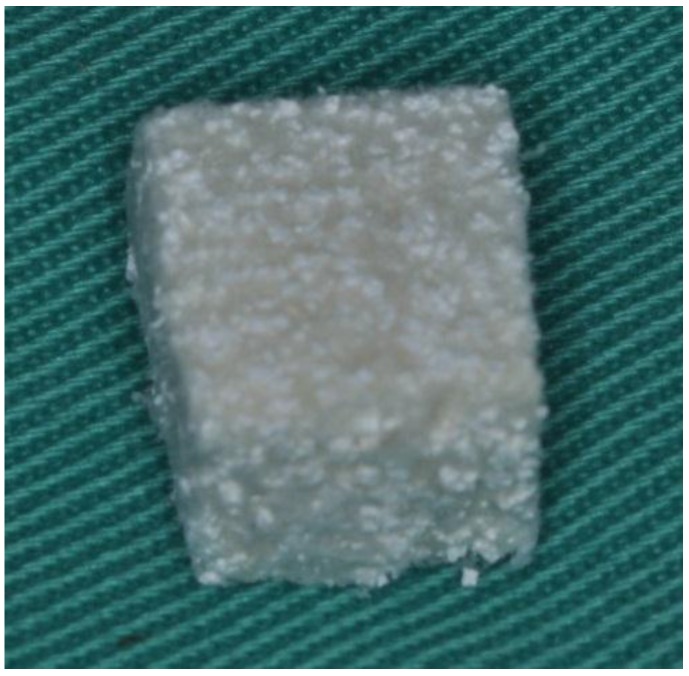
Hydroxyapatite/β-tricalcium phosphate-collagen composite.

The materials tested in this study comprised collagen plugs (CollaPlug^®^; Zimmer Dental Co., NewYork, NY, USA), bovine graft-collagen composite (Geistlich Bio-Oss Collagen^®^, Geistlich Co., Princeton, NJ, USA) and HA/β-TCP-collagen composite. The bovine graft-collagen composite was a cube consisting of Geistlich Bio-Oss^®^ bovine cancellous bone granules with 10% highly purified porcine collagen. The HA/β-TCP-collagen composite was a homogeneous plug consisting of purified porcine type I collagen fibers and HA/β-TCP ceramic with a weight ratio of 30:70, respectively. The HA/β-TCP used in the mixture had a hydroxyapatite/β-tricalcium phosphate ratio of 60:40. The mixture was poured into a plug mold and freeze-dried to yield HA/β-TCP–collagen composite plugs (Figure 10).

### 4.1. Scanning Electron Microscope Examination and Energy Dispersive Spectrometry

Elemental analyses of the collagen matrix distribution with the HA/β-TCP granules samples were performed using an electron microscope equipped with an energy dispersive X-ray spectrometer (EDS) (Model 2400; Hitachi, Ltd, Tokyo, Japan). This test was done with the purpose of studying the material surface and calcium-phosphate molar ratio.

### 4.2. Compression Strength

Compression strength of the hydroxyapatite/β-tricalcium phosphate (HA/β-TCP)-collagen composite composites was performed using a universal testing machine (AGS-1000D, Shimadzu, Tokyo, Japan) at a crosshead speed of 0.5 mm/s. After the examination, the compression strength were calculated.

### 4.3. Vitality Analysis

Cell metabolic activity was evaluated by succinic dehydrogenase (SDH) activity, which is a measure of the mitochondrial respiration of the cells. The results were read on a multiwell scanning spectrophotometer (Plate Chameleon Multilabel Detection Platform; HIDEX, Turku, Finland) and showed a high degree of precision. No washing steps were used in the assay. The main advantages of the colorimetric assay are that it is rapid, precise and lacks any radioisotope [29].

The composites were immersed in PBS solution and incubated in a humidified incubator at 37 °C; after 24 h, the PBS was removed and conserved. Later, MG-63 (ATCC CRL-1427) osteoblast-like cells, at a concentration of 1 × 10^4^ cells/mL, were cultured in 24-well plates (Costar Corp., Cambridge, MA, USA) seeded into the PBS, previously subtracted from the composites and incubated under 5% CO_2_ and 95% air at 37 °C for 24 h as the positive control group. MG-63 cells cultured with DMSO under 5% CO_2_ and 95% air at 37 °C were the negative control group. Tetrazolium salt (MTT kit, Roche Applied Science, Mannheim, Germany) was added and metabolically reduced to colored formazan by mitochondrial dehydrogenase in viable cells after 4 h of incubation according to the manufacturer’s instructions. After solubilizing, the formazan dye was added with 500 μL dimethyl sulfoxide for 5 min; the optical density of the medium was then determined by ELISA reader (Model 2020, Anthos Labtec Instruments, Eugendorf, Wals, Austria) at a wave length of 570 nm.

### 4.4. Surgical Model

This study was approved by the Animal Care and Ethics Committee of Taipei Level Biotechology Inc., IACUC 100901, Taipei, Taiwan, and all experiments were performed in accordance with the guidelines laid down by the U.S. National Institutes of Health (NIH) regarding the care and use of animals for experimental procedures and the European Communities Council Directive of 24 November 1986 (86/609/EEC).

In this study, twenty 6-month-old adult English beagles, which were obtained from Beagle Dog Incubation Center, National Pintung University of Science and Technology, Pintung, Taiwan, were used as the animal model. Each animal was held in a 1 m width × 1 m length × 1.2 m height cage under 25 °C. A soft diet and water were supplied twice a day. At the surgery, general anesthesia consisted of subcutaneous injection of 1 mL/kg atropine sulfate, followed by intramuscular injection of 0.20 mL/kg Zoletil 50 (Virbac Co., Carros, France). Routine dental infiltration anesthesia was used at each surgical site. After local injection of 2% lidocaine (3M-ESPE, Neuss, Germany), an impression of each dog’s mandible was taken, and a stone model cast was fabricated. Then, each dog received an intrasulcular incision in the left and right first, second, third and fourth mandibular premolars, to later on be carefully extracted using forceps without elevation of a muco-periosteal flap or compromising the marginal gingiva. After extraction, collagen plugs were implanted into the first premolar sockets in the mandibular area in Group I. The bovine graft-collagen composite was implanted into the mandibular second premolar sockets in Group II. The HA/β-TCP-collagen composite was implanted into the mandibular third premolar sockets in Group III. The mandibular fourth premolar extraction socket was left empty in Group IV, the control group (Table 3). Following material placement, just on the sockets with grafted materials, a muco-periosteal flap was elevated into the vestibule using a crestal incision and two vertical releasing incisions [43]. Flaps were coronally re-adapted achieving graft materials’ partially coverage and sutured using interrupted biodegradable sutures. After the surgery, an amoxicillin 125 mg and ketoprofen 25 mg prescription were given to the animals twice a day, and gentle brushing was done once a day for 7 days.

**Figure 11:**
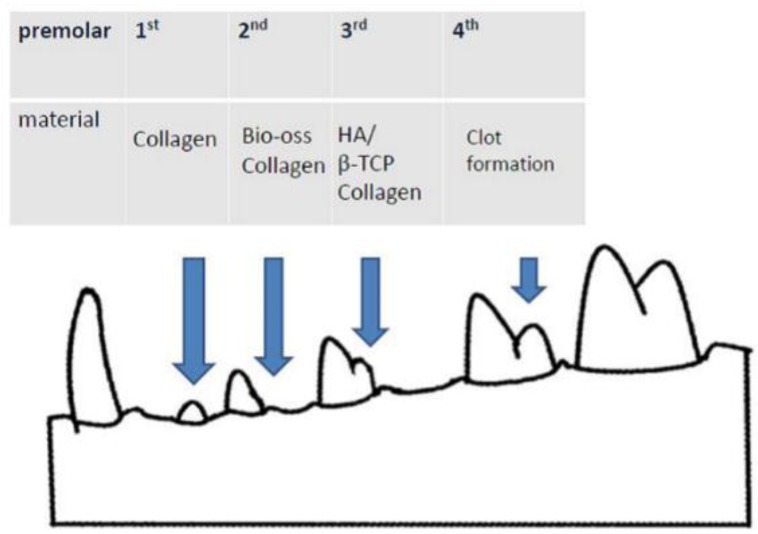
Surgical stent for the beagle’s mandible.

Impressions were taken, and dental gypsum models were cast before surgery and after 1, 2, 4 and 8 weeks of healing to replicate mandibular AR morphology. The width and height of the alveolar bone were measured on these dental gypsum models using an acrylic stent fabricated on the basis of the model before surgery (Figure 11).

Surgical stents, which used mandible canine and molar as guiding reference points with a guiding tube, were made bilaterally for each beagle.

### 4.5. Histological and Histomorphometry Analysis

The dogs were sacrificed at 1, 2, 4 and 8 weeks after surgery by intravenous injection of concentrated sodium pentobarbital. The samples and surrounding tissues were obtained and immediately immersed in 4% buffered formaldehyde (pH 7.4) for fixation. The samples were decalcified by 10% formic acid for 30 days, dehydrated in ethanol and then embedded in 10 × 10-mm paraffin blocks. Specimens were then cut into serial 4-μm sections, as described by Behnia *et al.* [44]. Two sections from the central area of each defect were selected, stained with hematoxylin and eosin (H & E) and subjected to histomorphometric analysis with Leica^®^ RM2255, Wetzlar, Germany and ImageJ software, developed by the NIH (Bethesda, MD) of the United States.

### 4.6. Statistical Analysis

Means and standard deviations of the horizontal and vertical dimensional changes of the alveolar bone were obtained for all samples in each group. The paired *t*-test was used to compare differences in the horizontal and vertical dimensional changes from Week 1 to Week 8 in each group. Because the interactions between the factors, treatment and dog, were low, the mean differences between dogs were not significant.

## 5. Conclusions

With the limitations of this study, it can be concluded that the HA/β-TCP-collagen composite is a novel biomimetic composite with osteoconductive properties. In this study, we showed that the HA/β-TCP-collagen composite is a viable bone substitute material in the horizontal dimension, both structurally and functionally, when used in socket preservation after tooth extraction.

## Figures and Tables

**Table:** 

Element	Wt %	At %
C K	50.95	67.16
O K	22.60	22.36
NaK	01.46	01.01
P K	07.03	03.59
MoL	02.71	00.45
PdL	02.39	00.36
CaK	12.86	05.08

**Table 1:** Horizontal dimensional changes after tooth extraction. Horizontal dimensional changes (in millimeters) of the alveolar bone implanted with collagen plugs, bovine graft-collagen composite, hydroxyapatite/β-tricalcium phosphate (HA/β-TCP)-collagen composite and the control group.

Horizontal (mm)				
Time	Collagen Plugs	Bio-Oss Collagen	HA/β-TCP-Collagen	Control
1 week	0.05 ± 0.05	0.17 ± 0.07	0.15 ± 0.10	0.01 ± 0.14
2 weeks	−0.11 ± 0.07	0.05 ± 0.08	0.14 ± 0.08	0.04 ± 0.05
4 weeks	−0.22 ± 0.05	−0.21 ± 0.10	−0.23 ± 0.08	−0.19 ± 0.09
8 weeks	−0.27 ± 0.12	−0.25 ± 0.07	−0.19 ± 0.09	−0.27 ± 0.18

**Table 2:** Vertical dimensional changes after tooth extraction. Vertical dimensional changes (in millimeters) of the alveolar bone implanted with collagen plugs, bovine graft-collagen composite, hydroxyapatite/β-tricalcium phosphate (HA/β-TCP)-collagen composite and the control group.

Vertical (mm)				
Time	Collagen Plugs	Bio-Oss Collagen	HA/β-TCP-Collagen	Control
1 week	−0.16 ± 0.15	−0.27 ± 0.07	−0.33 ± 0.13	−0.31 ± 0.12
2 weeks	−0.24 ± 0.17	−0.37 ± 0.07	−0.38 ± 0.08	−0.29 ± 0.12
4 weeks	−0.32 ± 0.10	−0.34 ± 0.28	−0.39 ± 0.12	−0.42 ± 0.28
8 weeks	−0.29 ± 0.21	−0.37 ± 0.16	−0.41 ± 0.24	−0.34 ± 0.12

**Table 3:** Study design for the beagle’s extraction socket. Different materials were placed into the different tooth extraction sockets. The control socket was empty.

Tooth	Materials
1st premolar	Collagen plug
2nd premolar	Bio-Oss collagen
3rd premolar	HA/-βTCP collagen
4th premolar	Empty
